# Predicting cardiovascular intensive care unit readmission after cardiac surgery: derivation and validation of the Alberta Provincial Project for Outcomes Assessment in Coronary Heart Disease (APPROACH) cardiovascular intensive care unit clinical prediction model from a registry cohort of 10,799 surgical cases

**DOI:** 10.1186/s13054-014-0651-5

**Published:** 2014-11-19

**Authors:** Sean van Diepen, Michelle M Graham, Jayan Nagendran, Colleen M Norris

**Affiliations:** Divisions of Critical Care and Cardiology, 2C2 WMC University of Alberta Hospital, 8440-112 St, Edmonton, AB Canada T6G 2B7; Division of Cardiology, 2C2 WMC University of Alberta Hospital, 8440-112 St, Edmonton, AB Canada T6G 2B7; Division of Cardiac Surgery, Mazankowski Alberta Heart Institute, 8440-112 St, Edmonton, AB Canada T6G 2B7; School of Public Health, University of Alberta, 116 Street and 85 Avenue, Edmonton, AB Canada T6G 2R3; Heart Health and Stroke Strategic Clinical Network, 8440 112 Street, Edmonton, AB Canada T6G 2B7

## Abstract

**Introduction:**

In medical and surgical intensive care units, clinical risk prediction models for readmission have been developed; however, studies reporting the risks for cardiovascular intensive care unit (CVICU) readmission have been methodologically limited by small numbers of outcomes, unreported measures of calibration or discrimination, or a lack of information spanning the entire perioperative period. The purpose of this study was to derive and validate a clinical prediction model for CVICU readmission in cardiac surgical patients.

**Methods:**

A total of 10,799 patients more than or equal to 18 years in the Alberta Provincial Project for Outcomes Assessment in Coronary Heart Disease (APPROACH) registry who underwent cardiac surgery (coronary artery bypass or valvular surgery) between 2004 and 2012 and were discharged alive from the first CVICU admission were included. The full cohort was used to derive the clinical prediction model and the model was internally validated with bootstrapping. Discrimination and calibration were assessed using the AUC c index and the Hosmer-Lemeshow tests, respectively.

**Results:**

A total of 479 (4.4%) patients required CVICU readmission. The mean CVICU length of stay (19.9 versus 3.3 days, *P* <0.001) and in-hospital mortality (14.4% versus 2.2%, *P* <0.001) were higher among patients readmitted to the CVICU. In the derivation cohort, a total of three preoperative (age ≥70, ejection fraction, chronic lung disease), two intraoperative (single valve repair or replacement plus non-CABG surgery, multivalve repair or replacement), and seven postoperative variables (cardiac arrest, pneumonia, pleural effusion, deep sternal wound infection, leg graft harvest site infection, gastrointestinal bleed, neurologic complications) were independently associated with CVICU readmission. The clinical prediction model had robust discrimination and calibration in the derivation cohort (AUC c index = 0.799; Hosmer-Lemeshow *P* = 0.192). The validation point estimates and confidence intervals were similar to derivation model.

**Conclusions:**

In a large population-based dataset incorporating a comprehensive set of perioperative variables, we have derived a clinical prediction model with excellent discrimination and calibration. This model identifies opportunities for targeted therapeutic interventions aimed at reducing CVICU readmissions in high-risk patients.

## Introduction

Coronary artery bypass grafting (CABG) and valvular surgery improve patient survival and quality of life [1-3]. In the United States alone, an estimated 242,000 patients underwent cardiac surgery in 2009 [4]. In many centers, postoperative cardiac surgical patients are admitted directly to a cardiovascular intensive care unit (CVICU) and, among patients discharged from the CVICU to lower acuity wards, a reported 2.3 to 7.8% require critical care unit readmission [5-9]. Importantly, these patients have longer lengths of CVICU stay, a higher risk of mortality, and have the potential to further limit institutional cardiac surgery capacity [6-8].

In medical and surgical intensive care units, risk factors for readmission have been identified and clinical risk prediction models have been published; however, considerably less information is available for the CVICU patient population [10-14]. Previous studies reporting CVICU readmission risk factors have been methodologically limited by a small number of outcomes, unreported measures of calibration or discrimination, lack of validation, or a lack of patient information spanning the entire perioperative period [5-8,15]. Identifying patients at high risk for CVICU readmission could potentially lead to interventions aimed at reducing unplanned CVICU readmission, surgical cancellations, and hospital costs.

Accordingly, using a comprehensive provincial registry with integrated patient, surgical, and postoperative data from coronary catheterization through to CVICU discharge, we sought to derive and internally validate a risk prediction model for CVICU readmission after cardiac surgery.

## Materials and methods

### Data sources

The Alberta Provincial Project for Outcomes Assessment in Coronary Heart Disease (APPROACH) is a registry of prospectively collected information on all patients undergoing cardiac catheterization and any subsequent cardiac procedures including cardiac surgery in the province of Alberta, Canada [16,17]. The registry captures detailed individual patient demographic, medical, angiographic, surgical, and postoperative information. Preoperative patient demographic and medical variables and well as cardiac diagnostic and surgical procedural information are entered into the dataset by trained cardiac catheterization laboratory and operating room staff at the time of the procedure. Postoperative CVICU variables and complications are extracted from patient charts by trained chart abstracters using Society of Thoracic Surgeons (STS) database data definitions to identify complications occurring before index ICU discharge [18]. Abstracters also review and extract all previously entered pre- and intraoperative variables. A notable exception to these STS definitions is that neurologic complications were defined as an aggregate of all STS neurologic complications including any cerebrovascular accident, intracranial hemorrhage, coma ≥24 hours, encephalopathy, or paralysis. The latter composite definition was employed because the APPROACH dataset did not code for individual neurologic complications, only the incidence of any STS-defined neurological complication. Mortality is tracked through an Alberta Bureau of Vital Statistics data linkage [16]. This study, and a waiver of patient consent in this prospectively collected data registry, was approved by the University of Alberta Health Research Ethics Board (Pro00042669).

### Study population

A total of 10,799 patients ≥18 years who underwent CABG and/or valvular surgery between January 2004 and December 2012 and were discharged from the CVICU alive from the University of Alberta were included in this analysis. Patients in our institution are admitted to the CVICU after CABG and valvular surgeries then discharged to a surgical postoperative ward when it was deemed clinically appropriate by the attending physician. For each surgical hospitalization, only patient, procedural, and postoperative variables recorded up to the time of the first CVICU discharge were evaluated in the prediction model. Excluded patients included: noncardiovascular surgical admissions, cardiac or pulmonary transplants, and isolated ventricular assist device or extracorporeal membrane oxygenation insertions.

### Outcomes of interest

The primary outcome of interest was defined as any critical care unit readmission after CVICU discharge during the index surgical admission. Secondary outcomes of interest included hospital length of stay, in-hospital mortality, and one-year mortality.

### Statistical analyses

Continuous variables were summarized using means and standard deviations and medians where appropriate. Categorical variables were summarized using percentages. Differences in categorical variables were tested using chi-square tests and differences in continuous variables were tested using Student *t* tests. The clinical prediction model was derived using the multivariable logistic regression model and CVICU readmission as the outcome variables. Independent variables of interest were entered in blocks. Individual covariates were entered and removed from the model with point estimates and model performance was evaluated with each step [19]. Perioperative covariates included in the model derivation included demographics (age, body mass index), medical history (hypertension, type 1 and type 2 diabetes, family history of heart disease, heart failure, New York Heart Association class, prior CABG, Canadian Cardiovascular Society class cerebrovascular disease, chronic liver disease, chronic lung disease, chronic renal disease, presurgical dialysis), preoperative coronary anatomy (one- and two-vessel disease, three-vessel disease, left main disease, not available), preoperative left ventricular ejection fraction (>50%, 35 to 50%, 20 to 34%, <20%, not available, not recorded due to hemodynamic instability), surgical priority (low risk, emergent, urgent in-hospital, urgent out of hospital), surgical procedure (isolated CABG, isolated valvular surgical repair or replacement, CABG and single-valve surgical repair or replacement, single-valve repair or replacement plus non-CABG surgery, multivalve repair or replacement), surgical incidence (first cardiac reoperation, ≥2 prior cardiac operations), cardiopulmonary bypass time, intraoperative red blood cell transfusion, intraoperative fresh frozen plasma, and postoperative CVICU complications (cardiac arrest, atrial fibrillation, cardiac tamponade, mechanical ventilation >24 hours, re-intubation, superficial sternal infection, deep sternal wound infection, sepsis, leg harvest site infection, urinary tract infection, acute respiratory distress syndrome, chest tube insertion, pneumonia, pleural effusion, positive sputum culture, pulmonary embolism, pulmonary edema, gastrointestinal bleed, mesenteric ischemia, renal failure, postoperative dialysis, delirium). The final derivative parsimonious model included all variables that remained independently significantly associated with CVICU readmission. For the purposes of validating the model, and estimating the population parameters, a bootstrapping technique was used. One thousand bootstrap samples with replacement were used to derive robust estimates of standard errors and confidence intervals for estimates of the odds ratio for all predictor variables that were independently associated with CVICU readmission. Scores were created by using the method described in the development of the Framingham risk scores [20]. Statistical analyses were performed using IBM SPSS statistics version 21 software (IBM Corp, Armonk, NY, USA).

## Results

The final study population consisted of 10,799 patients. A total of 479 (4.4%) patients were discharged alive and then readmitted to an intensive care unit. Among the readmitted patients, 91.7% were readmitted once, 7.2% were readmitted twice, and 1.1% were readmitted three or more times. Clinical preoperative characteristics among patients with and without an intensive care unit readmission are presented in Table 1. Readmitted patients were more likely to be older, have a history of hypertension, type 2 diabetes, a prior CABG, heart failure, chronic lung disease, chronic liver disease, and chronic kidney disease. The extent of coronary artery disease was similar between the two groups; however, readmitted patients were more likely to have lower preoperative ejection fractions.

**Table 1:** **Clinical characteristics of cardiac surgical patients with and without a cardiovascular intensive care unit readmission**

**Characteristic**	**No readmission (n = 10,320)**	**Readmission (n = 479)**	***P*** **value**
**Demographics**			
Age, mean (SD), years	64.6 (12.7)	68.9 (12.9)	<0.001
Male, %	75.8	71.0	0.17
BMI, mean (SD), kg/m^2^	28.9 (5.9)	28.5 (5.5)	0.112
**Medical history, %**			
Hypertension	77.5	81.6	0.035
Dyslipidemia	89.7	88.3	0.315
Type 1 diabetes	1.0	1.9	0.063
Type 2 diabetes	29.4	35.9	0.002
Prior myocardial infarction	45.1	45.5	0.86
Prior PCI	14.2	13.2	0.537
Prior CABG	3.0	6.5	<0.001
Heart failure	15.7	29.0	<0.001
NYHA class			
Class I	7.0	4.0	<0.001
Class II	13.9	12.1	
Class III	15.6	19.4	
Class IV	4.3	7.5	
Not entered	59.1	57.0	
Cerebrovascular disease	12.5	17.5	0.001
Current smoker	24.7	23.4	0.525
Chronic lung disease	32.6	52.4	<0.001
Chronic liver disease	1.0	3.5	<0.001
Chronic renal failure	2.2	5.8	<0.001
Preoperative dialysis	1.4	2.9	0.005
**Preoperative investigations**			
Extent of coronary artery disease (≥70%), %			0.452
0	17.9	18.4	
1 or 2	11.9	14.0	
3	38.6	34.9	
Left main	21.9	22.3	
Not available	9.7	10.4	
Left ventricular ejection fraction, %			<0.001
>50%	35.3	23.2	
>35-50%	17.1	14.8	
>20-34%	4.2	7.5	
<20%	1.0	3.3	
Not done due to emergency surgery	15.9	21.3	
Not available	26.5	29.9	
Hemoglobin, mean (SD), g/L	136.3 (18.5)	128.0 (20.8)	<0.001
Creatinine, mean (SD), μmol/L	100.6 (65.4)	116.4 (81.1)	<0.001

BMI: body mass index; CABG: coronary artery bypass graft; NYHA: New York Heart Association; PCI: percutaneous coronary intervention; SD: standard deviation.

The baseline surgical differences are provided in Table 2, and postoperative complications in Table 3. Unadjusted CVICU readmission was more common in patients undergoing emergent or urgent in-hospital surgery, cardiac re-operation, CABG plus valvular surgery, single-valve repair or replacement plus non-CABG surgery, or the repair or replacement of ≥2 valves. Similarly, postoperative pulmonary, cardiac, infectious, neurologic, gastrointestinal, and renal complications were all higher among patients with a CVICU readmission.

**Table 2:** **Operative variables in patients with and without a cardiovascular intensive care unit readmission**

**Operative variable**	**No readmission (n = 10320)**	**Readmission (n = 479)**	***P*** **value**
**Surgical priority, %**			0.009
Emergent	3.6	5.0	
Urgent in-hospital	40.0	46.3	
Urgent out of hospital	47.8	43.4	
Nonurgent out of hospital	8.6	5.2	
**Surgical incidence, %**			0.01
First operation	91.4	86.8	
Second operation	7.0	11.3	
Third or greater	1.6	1.9	
**Surgery, %**			<0.001
Isolated CABG	49.2	32.6	
CABG and single valve	7.7	12.1	
Isolated aortic valve repair or replacement	6.3	5.8	
Isolated mitral valve repair or replacement	2.4	1.9	
Multivalve repair or replacement	1.5	3.3	
**Intraoperative variables**			
Cardiopulmonary bypass time, mean (SD) min	127.1 (67.2)	149.0 (56.2)	<0.001
Aortic cross-clamp time, mean (SD), min	88.8 (55.8)	103.4 (48.8)	<0.001
Intraoperative RBC transfusion, %	25.7	50.3	<0.001
Intraoperative FFP transfusion, %	12.3	27.1	<0.001

CABG: coronary artery bypass graft; FFP: fresh frozen plasma; RBC: red blood cell; SD: standard deviation.

**Table 3:** **Postoperative complications in patients with and without a cardiovascular intensive care unit readmission**

	**No readmission (n = 10,320)**	**Readmission (n = 479)**	***P*** **value**
**Pulmonary complications**			
Prolonged mechanical ventilation (>24 hours)	18.4	57.3	<0.001
Total mechanical ventilation, hours	22.5	92.9	<0.001
Re-intubation	2.7	48.2	<0.001
Acute respiratory distress syndrome	3.1	33.3	<0.001
Pleural effusion	6.1	28.8	<0.001
Chest tube insertion	4.1	16.0	<0.001
Pulmonary embolism	0.2	1.3	0.001
**Cardiac complications**			
Cardiac tamponade	1.7	11.1	<0.001
Re-operation	3.3	16.9	<0.001
Cardiac arrest	1.7	17.3	<0.001
Heart block	2.0	7.5	<0.001
Atrial fibrillation	26.9	48.6	<0.001
**Infectious complications**			
Superficial sternal wound infection	3.6	9.2	<0.001
Deep sternal wound infection	0.4	8.1	<0.001
Leg venous harvest site infection	2.9	10.9	<0.001
Pneumonia	12.0	53.3	<0.001
Urinary tract infection	0.7	4.6	<0.001
Sepsis (any source)	1.5	16.5	<0.001
**Neurologic complications**			
Neurologic complication^a^	1.5	7.5	<0.001
Delirium	2.5	15.7	<0.001
**Gastrointestinal complications**			
Bleeding	1.0	14.8	<0.001
Mesenteric ischemia	0.5	4.4	<0.001
**Renal complications**			
Renal failure	5.9	31.3	<0.001
Postoperative dialysis	2.1	18.6	<0.001

^a^Neurologic complication defined as: cerebrovascular accident, intracranial hemorrhage, coma ≥24 hours, encephalopathy, or paralysis.

Cardiac surgical patients with a critical care unit readmission had significantly longer hospital (41.7 days vs. 9.3 days, *P* <0.001) stays. The median (3.0 vs. 1.0 days, *P* <0.001) and mean (7.36 vs. 2.66 days, *P* <0.001) initial lengths of CVICU stays were longer in the readmission group. The total number of intensive care unit days was 19.9 days in the readmission cohort. The in-hospital (14.4% vs. 2.2%, *P* <0.001) and one-year mortality (21% vs. 4.2%, *P* <0.001) rates were also higher in readmission patients.

### Predictors of readmission

**Figure 1:**
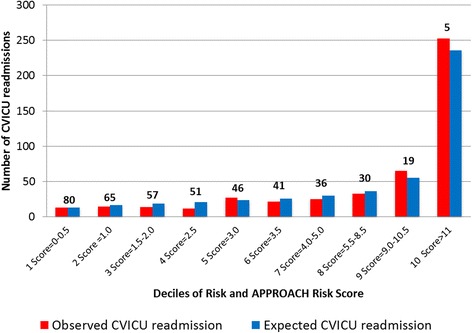
**Observed versus predicted probability of all-cause CVICU readmission and number needed to identify (NNI) one readmission across risk deciles.** The number needed to identify (NNI) one readmission is lower in higher readmission risk deciles. CVIVU: cardiovascular intensive care unit.

A total of 12 variables were independent predictors of intensive care unit readmission after cardiac surgery (Table 4). The final model included four preoperative variables (age ≥70 years, chronic lung disease, ejection fraction (EF) 35 to 50%, EF 20 to 34%, and EF <20%), two intraoperative variables (single-valve repair or replacement plus non-CABG surgery, multivalve repair or replacement), and seven postoperative complications (cardiac arrest, pneumonia, pleural effusion, deep sternal wound infection, leg graft harvest site infection, gastrointestinal bleed, and neurologic complications). The percentage of the total adjusted Wald χ2 for pre-, intra-, and postoperative independent variables were 13.8%, 3.1%, and 83.1%, respectively. The model demonstrated robust discrimination (c index = 0.799) and calibration (Hosmer-Lemeshow χ2 = 11.17, *P* = 0.192; Figure 1). The relationship between observed and expected readmission to intensive care across risk deciles is shown in Figure 1. The number of screened patients needed to identify (NNI) one readmission was calculated *post hoc* across each risk decile and the results were as follows: decile 1 (score 0 to 0.5, NNI = 80), decile 2 (score 1.0, NNI = 65), decile 3 (score 1.5 to 2.0, NNI = 57 ), decile 4 (score 2.5, NNI = 51 ), decile 5 (score 3.0, NNI = 46 ), decile 6 (score 3.5, NNI = 41), decile 7 (score 4.0 to 5.0 NNI = 36), decile 8 (score 5.5-8.5, NNI = 30), decile 9 (score 9.0 to 10.5, NNI = 19), decile 10 (score >11, NNI = 5).

**Table 4:** **Perioperative variables independently predictive of cardiovascular intensive care readmission**

**Variable**	**Derivation cohort**		**Internal validation**
	**Wald** χ^**2**^	**OR (95% CI)**	**OR (95% CI)**
**Patient variables**			
Age ≥70 years, per 10 years	20.56	1.23 (1.11, 2.66)	1.23 (1.12, 1.36)
Chronic lung disease	14.40	1.49 (1.21, 1.83)	1.49 (1.21,1.87)
**Ejection fraction (EF)** ^**a**^			
EF 20-34%	6.03	1.64 (1.11, 2.44)	1.64 (1.05,2.37)
EF <20%	13.54	3.06 (1.69, 5.55	3.06 (1.49,5.35)
**Surgical variables**			
Single-valve repair or replacement + non-CABG surgery^b^	5.86	1.41 (1.07, 1.86)	1.41 (1.05,1.82)
Repair or replacements of ≥2 valves^b^	6.2	2.13 (1.18, 3.86)	2.13 (1.00,3.88)
**Postoperative CVICU variables**			
Cardiac arrest	65.06	4.04 (2.88, 5.66)	4.03 (2.69, 6.15)
Pneumonia	77.84	3.08 (2.40, 3.96)	3.08 (2.37, 4.09)
Pleural effusion	45.65	2.86 (2.11, 3.88)	2.86 (2.03, 3.83)
Deep sternal wound infection	48.29	6.58 (3.87, 11.18)	6.57 (3.33,13.11)
Leg graft harvest site infection	4.93	1.56 (1.05, 2.31)	1.56 (1.02,2.46)
Gastrointestinal bleed	61.45	4.67 (3.18, 6.86)	4.66 (2.93,7.22)
Neurologic complication^c^	24.16	2.22 (1.61, 3.05)	2.21 (1.57, 3.17)

^a^Reference ejection fraction >50%; ^b^reference isolated coronary artery bypass; non-CABG procedures most commonly included left atrial appendage ligations, maze procedures, and atrial septal defect or patent foramen ovale closures; ^c^neurologic complication defined as: cerebrovascular accident, intracranial hemorrhage, coma ≥24 hours, encephalopathy, or paralysis. CABG: coronary artery bypass grafting; CI: confidence interval; CVICU: cardiovascular intensive care unit; OR: odds ratio.

The stability of the 12 model variables was assessed in 1,000 bootstrap samples. We used the bootstrapping method to derive robust estimates of the standard errors of the odds ratios of the variables in the derivation model. All variables in the derivation model were retained in the bootstrapped model (Table 4). The confidence intervals estimated by the bootstrapped samples showed that the variables in the derivation model appropriately reflect the statistically independent predictors of the probability of readmission to the CVICU.

### Risk score

**Figure 2:**
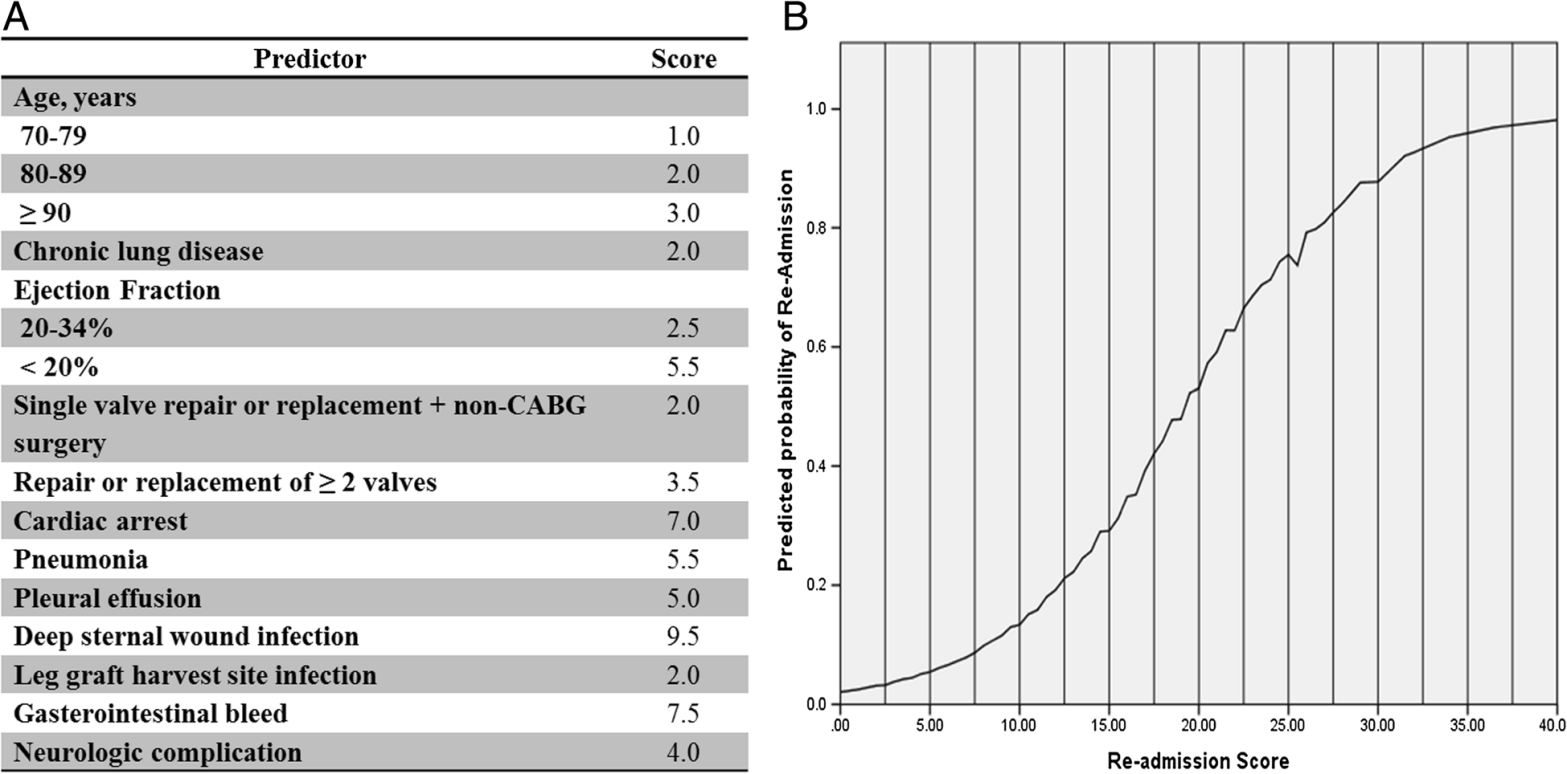
**The APPROACH CVICU readmission risk model nomogram. (A)** The CVICU readmission risk score and **(B)** mean predicted CVICU readmission by APPROACH CVICU readmission risk score. APPROACH: Alberta Provincial Project for Outcomes Assessment in Coronary Heart Disease; CVICU: cardiovascular intensive care unit.

The perioperative independent variables in the multivariable model were used to create the APPROACH CVICU readmission score (Figure 2A). The relationship between risk score and the predicted probability of intensive care readmission is also shown in Figure 2B which shows a corresponding rise in the predicted risk of CVICU readmission as the APPROACH CVICU risk score rises.

## Discussion

This study using a large cohort of cardiac surgical patients with detailed documentation of perioperative clinical information and postoperative complications has several important findings. First, intensive care unit readmission after CVICU discharge is associated with significantly longer hospital length of stay and mortality. Second, intensive care readmission is predictable with more than 83% of total adjusted risk derived from postoperative complications. Third, we derived and validated a clinical prediction score with excellent discrimination and calibration for intensive care readmission in cardiac surgical patients using variables spanning the entire perioperative period.

Cardiovascular surgery mortality has declined over recent decades despite an increase in high-risk older surgical patients [21,22]. Up to 8% of all cardiac surgical patients undergoing CABG or valvular surgery will be readmitted to an intensive care unit and, importantly, cohort studies have reported that in-hospital mortality rates of 11 to 31% in readmitted patients [5-8]. The present study confirms the risks of mortality and length of stay with intensive care readmission and builds on these findings with one-year outcomes suggesting that intensive care readmission is a risk for long-term mortality among readmission survivors.

Multiple studies have reported clinical variables associated with intensive care readmission after CVICU discharge; however, the clinical applicability of these models has been limited by a lack of model calibration or discrimination measures, validation, and/or patient information spanning the entire perioperative period [5-9]. In our model, most of the risk (88%) of readmission was due to postoperative complications. This finding suggests that contemporary operative mortality scores based largely on preoperative clinical indices are ill-suited for readmission prediction and that development of a readmission-specific prediction model is required [23,24].

Among the pre- and intraoperative clinical variables predictive of readmission in this model, age, lung disease, left ventricular ejection fraction, and surgical procedure have been reportedly associated with readmission in previous studies [6-9]. Notably, in our derivation cohort the point estimate for readmission risk was lower among patient with an ejection fraction less than 20% compared to patients with an EF 20 to 34%. Although EF is a recognized readmission risk factor, our finding that very low ejections fractions are associated with additional risk may be novel. From a comprehensive set of postoperative variables and complications, seven variables were predictive of readmission in this model. Previous studies have shown that respiratory complications are independently associated with readmission while infectious complications, stroke, intracranial hemorrhage, and gastrointestinal bleeding have been reported to increase postoperative mortality [8,25-29]. Postoperative cardiac arrest, to our knowledge, has not been reported as an independent risk factor for CVICU readmission, though it is associated with higher in-hospital mortality and longer hospital stays [30-33].

The readmission model demonstrated excellent discrimination and calibration. We hypothesize that this robust model performance is due to the inclusion of a comprehensive set of clinical, surgical and postoperative variables recorded up to the first CVICU discharge. Moreover, the accuracy of the APPROACH CVICU score makes it an attractive potential tool for future clinical and quality improvement research. Previous studies have reported that the implementation of intensivist-led medical emergency team follow-up after discharge from general intensive care units significantly reduced intensive care readmission; however, the value of targeted follow-up in high-risk postcardiac surgical patients remains unclear [34,35]. Exploring the outcomes associated with targeted post-CVICU discharge follow-up by either cardiology for heart failure patients or medical emergency teams for patients with noncardiac postoperative complications at high risk for readmission are important potential applications of this model.

### Limitations and strengths

The limitations of this analysis merit consideration. First, the study only included patients undergoing CABG and valvular surgeries; thus, the prediction model may not apply to patients undergoing other cardiac surgical procedures. The model was constructed on the most common cardiac surgeries in an effort to maximize external generalizability because procedures such as transplants, ventricular assist devices, and adult congenital surgeries are often conducted only in specialized centers. Second, the model requires validation in an external dataset; however, an internal validation with bootstrapping was applied to the derivation model. Third, no CVICU physiologic data or postoperative medication data were available in this dataset, though it should be noted that the discrimination and calibration of the model were excellent with available variables. Fourth, although the postoperative complications were extracted by trained abstracters using standardized definition into a registry module that codes for CVICU complications the dataset’s coding has not been independently validated.

## Conclusions

In a large prospective observational dataset of unselected patients undergoing CABG and/or valvular surgery, we developed and validated a clinical prediction model for intensive care unit readmission after CVICU discharge using a comprehensive set of perioperative variables that demonstrated excellent discrimination and calibration. The APPROACH CVICU readmission score can be used to identify high-risk patients at the time of CVICU discharge and it may provide future opportunities for targeted therapeutic interventions aimed at reducing CVICU readmissions.

## Key messages

- Using a comprehensive set of perioperative variables, a clinical prediction model with excellent discrimination and calibration was derived and validated.
- A total of three preoperative, two intraoperative, and seven postoperative variables were predictive of CVICU readmission.
- This score may provide future opportunities for targeted therapeutic interventions aimed at reducing CVICU readmission.

## Abbreviations

APPROACH: Alberta Provincial Project for Outcomes Assessment in Coronary Heart Disease
CABG: coronary artery bypass grafting
CVICU: cardiovascular intensive care unit
EF: ejection fraction
NNI: number needed to identify
STS: Society of Thoracic Surgeons
